# Effect of Virtual Reality on Pain and Anxiety During Epidural Steroid Injection in Patients with Lumbar Radicular Pain: An Open-Label Randomized Trial

**DOI:** 10.3390/healthcare13182376

**Published:** 2025-09-22

**Authors:** Marine Javelot, Clément Chopin, Loïs Bolko, Ambre Hittinger, Marion Geoffroy, Isabelle Charlot, Fanny Adeline, Claire Coutureau, Alice Duvivier, Jean-Hugues Salmon

**Affiliations:** 1 Department of Rheumatology, University Hospital of Reims, 51100 Reims, France; cchopin@chu-reims.fr (C.C.); lbolko@chu-reims.fr (L.B.); ahittinger-roux@chu-reims.fr (A.H.); marionduguit@gmail.com (M.G.); icharlot@chu-reims.fr (I.C.); fanny.adeline@laposte.net (F.A.); jhsalmon@chu-reims.fr (J.-H.S.); 2 Departement of Rheumatology, University of Reims Champagne Ardenne (URCA), 51100 Reims, France; 3 Department of Clinical Research and Public Health, University Hospital of Reims, 51100 Reims, France; ccoutureau@chu-reims.fr (C.C.); alicedvvr14@gmail.com (A.D.)

**Keywords:** virtual reality, pain, anxiety, lumbar radicular pain, glucocorticoid injection

## Abstract

**Background/Objectives**: Virtual reality (VR) has been shown to reduce pain and anxiety in several specialties, but has not been investigated in the setting of steroid injections in rheumatology. We aimed to assess the impact of using a VR headset on pain and anxiety during epidural steroid injection via the sacral hiatus for lumbar radiculopathy. **Methods**: Patients received two injections via the sacral hiatus and were randomized into one of two groups: group 1 used the VR headset during the first injection and not during the second injection, while group 2 used the VR headset during the second injection but not the first. The primary endpoint was pain evaluated on a numeric rating scale. Secondary objectives were anxiety, measured using the STAI (State Trait Anxiety Inventory), and safety. These analyses were performed using the Mann–Whitney U test. **Results**: We included 116 patients over 18 years of age who were hospitalized in the Rheumatology department of the University Hospital of Reims and scheduled to receive at least two epidural steroid injections. We observed a significantly lower pain score during the first injection procedure (median 3 (IQ 1; 6) in group 1 vs. 5 (IQ 3; 7) in group 2, *p* = 0.045). The analysis for the second injection could not be performed by intention-to-treat due to the presence of a sequence effect. There was also a significant reduction in anxiety (*p* = 0.004 and *p* = 0.002 by per-protocol analysis). **Conclusions**: VR can significantly reduce pain and anxiety during epidural steroid injection via the sacral hiatus.

## 1. Introduction

Virtual reality (VR) is a technology that has garnered increasing traction in healthcare since the 1990s [1]. It has been proven useful for reducing pain and anxiety in pediatric patients [2], for pain control [3], and in the settings of oncology [4] and obstetrics [5]. Recently, but outside of our specialty, our cardiology colleagues have used virtual reality headsets during coronary catheterization procedures. Virtual reality therapy was not non-inferior to pharmacological sedation for reducing pain during coronary angiography or angioplasty [6]. The utility of VR has previously been studied in the setting of rheumatology, where it was shown to enable strength–balance training with a view to reducing fear of falling in primary osteoporotic women [7]. It has been shown to improve pain, function, and quality of life in patients with knee osteoarthritis [8] and chronic lower back pain [9,10]. Finally, VR was also shown to improve cardiopulmonary capacity and quality of life via VR exercise in patients with fibromyalgia [11].

The lifetime prevalence of lumbar radiculopathy is between 13 and 40% [12]. Lumbar spinal injections are among the key treatments proposed for mechanical spinal pathologies [13,14]. Among the various approaches possible (transforaminal, interlaminar, or sacrococcygeal hiatus), caudal epidural injection via the sacral hiatus is widely used because there is less risk of puncturing the dura. Furthermore, a prior history of spinal surgery is not a contraindication of caudal epidural injection. Overall, the safety profile of caudal epidural injection is good, with the most common side effects being vasovagal reaction, anxiety, increased radicular pain, and injection site pain [13]. The procedure is mostly performed without premedication, but the equimolar oxygen–nitrous oxide 50%/50% mixture (known in French under the acronym “MEOPA”) can be used to reduce pain and anxiety [15]. However, MEOPA is not easy to implement for rheumatologists in private practice, and its use is also not devoid of adverse effects [16,17].

There is a paucity of data regarding the use of virtual reality headsets, particularly within the field of rheumatology. Therefore, the aim of this study was to evaluate the impact of VR on pain and anxiety during epidural steroid injection via the sacral hiatus in patients with lumbar radiculopathy.

This publication has been modified to improve its clarity and relevance compared to the abstracts [18,19] published in conference proceedings. Details on the injection procedure, as well as explanations of the methodologies and results, have been provided.

## 2. Materials and Methods

We performed a single-center, randomized, open-label, interventional, superiority trial using a cross-over design at the University Hospital of Reims in Northern France, from February to August 2023.

### 2.1. Study Population

Adult patients (aged ≥ 18 years) who were hospitalized in the Rheumatology department of the University Hospital of Reims and scheduled to receive at least two epidural steroid injections to treat lumbar radiculopathy were eligible to participate. Two injections are the usual practice in the department. It is recommended to perform repeated injections in patients with intense pain [20] for a medium-term effect [21]. Non-inclusion criteria were as follows: premedication with nitrous oxide or painkillers or anxiolytics; patients with visual, auditory, or vestibular deficits; severe cognitive impairment; a history of epilepsy; claustrophobia; patients under any form of judicial or legal protection (such as tutorship); and patients with psychotic disorders.

### 2.2. Study Design

The patients were recruited and enrolled by the physicians of the Rheumatology department of the University Hospital of Reims. The randomization list was generated using the software CleanWeb Version 175.4.1 (Telemedicine Technologies, Boulogne-Billancourt, France) in a 1:1 ratio and in blocks. After providing written informed consent, patients were randomly assigned to one of two groups, as follows:

Group 1: Patients used the VR headset during the first epidural injection, but not during the second injection.

Group 2: Patients used the VR headset during the second epidural injection, but not during the first injection.

On the day of randomization (day 1), patients were invited to fill out a questionnaire to record their socio-demographic data and other data (pain level, symptom duration, first injection, STAI before the injection, use of anxiolytics or antidepressants, if they have ever tried hypnosis or sophrology, and the choice of the environment). Then, the first epidural injection procedure was performed by a physician expert injection, with or without the VR headset depending on the randomization group.

We performed the injection into the sacral hiatus. The sacral hiatus is bordered laterally by the sacral cornua and represents a natural defect in the dorsal midline union, forming the caudal end of the central spinal canal and opening into the epidural space.

We injected 5 mL of HYDROCORTANCYL 2.5% and 10 mL of saline solution 0.9% using a 21G (0.80 mm) needle, after clinically locating the sacrococcygeal hiatus. No ultrasound guidance was used, and no local anesthetic was infiltrated.

At the end of the first injection procedure, patients assessed their pain during the injection (related by the injection) using a verbal numeric rating scale (VNRS) ranging from 0 to 10 [22]. Anxiety was assessed using the validated State Trait Anxiety Inventory (STAI) instrument [23].

On day 2, there were no study procedures of any sort, and patients rested.

On day 3, the second epidural injection was performed by the same physician, with or without the VR headset depending on the randomization group. At the end of the procedure, patients once again evaluated the pain and anxiety they felt during the procedure, as detailed above for day 1.

Both injections were performed by the same experienced operator for all patients.

The cross-over design was adopted to reduce the number of patients required, and also with the consideration that it might influence efficacy. The concept of the patient serving as their own control is particularly compelling.

The VR headset was provided by HealthyMind (Paris, France) and is considered as a CE mark class 1 medical device. The VR headset used was a Pico G2 4K, model A7510, connected to Bose Soundlink 2 AE headphones. Both the headset and earphones were connected to a tablet that could be used to choose the VR environment, with a choice between a Zen garden, snowy mountain, sunny mountain, beach, underwater, and forest environment. In parallel, hypnosis exercises were also available (proposed by the medical device), and each patient was free to do them or not.

The full duration of participation of each patient was 3 days as usual in the unit (randomization and first injection on day 1, rest on day 2, second injection on day 3). After discharge, patients were followed by their usual physician (e.g., the general practitioner or rheumatologist who addressed the patient to our unit) according to usual practice.

### 2.3. Outcomes

- The primary endpoint was the pain score during the injection, as assessed by a numeric rating scale ranging from 0 to 10 [22].
- Secondary endpoints were anxiety during the injection procedure, as assessed by the STAI, level of satisfaction with the use of the VR headset (measured on a scale from 0 to 10), and safety (occurrence of any one or more adverse events from among the following: discomfort; asthenia; sleepiness; headache; vertigo; difficulty concentrating; nausea; eye fatigue; eye pain; visual fatigue; blurred vision). These adverse events were commonly described in the literature. Furthermore, satisfaction was measured on a scale from 0 to 10 (not satisfied to very satisfied). It was a bespoke measurement.

### 2.4. Statistical Analysis

The sample size of 116 patients was calculated on the basis of an expected 2-point decrease [24] (SD ± 2.82 group 1; SD ± 2.55 group 2) in VNRS, with power of 80% and 15% missing data. *p* values < 0.05 were considered statistically significant. These analyses were performed using the Mann–Whitney U test. Analyses were performed using SAS software version 9.4 (SAS Institute Inc., Cary, NC, USA). Quantitative data are described as median and interquartiles (IQR) (due to non-normal distributions), and qualitative data are described as numbers and percentage. Data were first analyzed on an intention-to-treat basis. When necessary, missing data were imputed with the worst observed value. In case of missing data, we assigned the worst result observed for a patient during the study. For example, if the worst observable STAI score in the study was 77, then the patient was assigned this value. Per-protocol analyses were also performed.

Data were analyzed with the usual statistical methods for cross-over trials with two groups and two periods. Firstly, interactions between period and device, and pain and anxiety were tested. If the interaction was significant, we assessed device efficacy during the first period only. Then, we tested the period effect to evaluate whether pain and anxiety at first epidural injection differed from those at the second injection. Finally, we evaluated the effect of the VR headset on pain and anxiety.

### 2.5. Role of the Funding Source

The study was approved by the Ethics Committee “CPP South East IV” on 3 August 2022 (under the number 2022-A00769-34). The study was recorded under number ID-RCB: 2022-A00769-34. The clinical trial was registered in ClinicalTrials.gov under number ID NCT05505968. This randomized clinical trial was conducted in accordance with the CONSORT 2010 guidelines. The CONSORT checklist is provided in the Supplementary Materials. Healthy Mind provided the VR headsets for the study, but the company was not involved in the writing of the protocol, or in the design, analysis, or interpretation of the results.

## 3. Results

### 3.1. Population of Study

**Figure 1:**
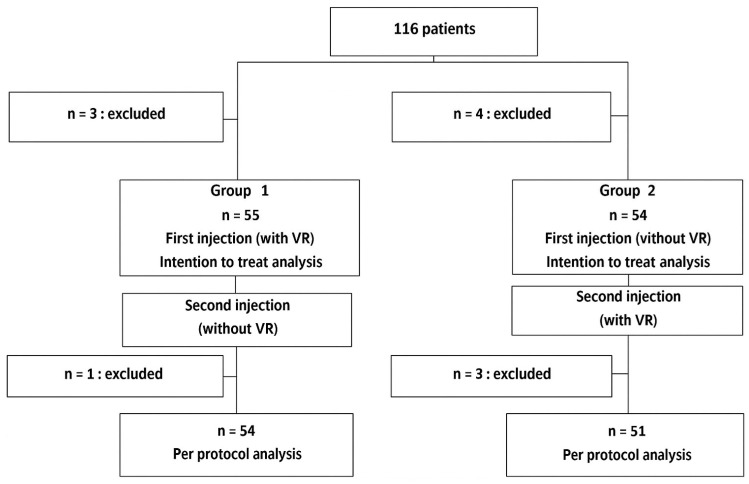
Study flow chart.

A total of 116 patients were included in the study from August 2022 to February 2023; 58 were in each group. The flowchart of the study inclusions is shown in (Figure 1).

Three patients were excluded before the first injection in group 1 (other injection which was not in the protocol, withdrawal of consent). Four patients were excluded before the first injection in group 2 (fungal infection, withdrawal of consent, counter argument to the injection procedure). In all, 109 patients were analyzed with an intention to treat.

One patient in each group had the VR at the wrong time, while two patients had important missing data and were excluded from per-protocol analysis. A total of 105 patients were analyzed with per-protocol.

The characteristics of the study population are shown in (Table 1).

Groups were comparable for all variables. Median age was 61 years in group 1 versus 54.5 years in group 2. There were more female patients in each group (56.3% in group 1 vs. 51.8% in group 2). Median duration of symptoms was 6 months. Median pain intensity was 6 and was similar in each group. For around 70% of patients, it was their first time being treated with epidural injection.

### 3.2. Primary Outcome

**Figure 2:**
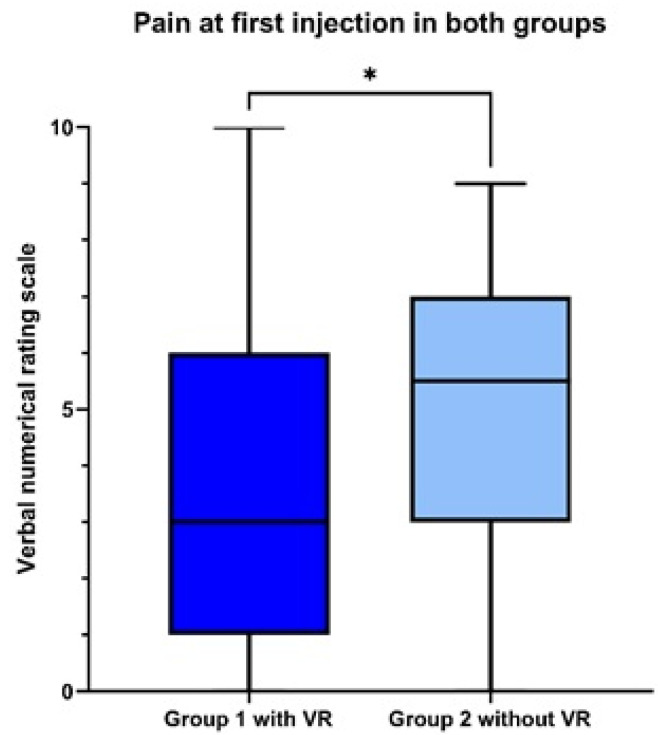
Primary outcome. Box plot illustrating the magnitude of pain reduction observed across the study groups. * significant difference.

Through intention-to-treat analysis, there was a significant reduction in pain scores with the use of the VR headset during the first injection procedure: median 3 [1,2,3,4,5,6] in group 1 vs. 5 [3,4,5,6,7] in group 2; (*p* = 0.045) (Figure 2).

The analysis for the second injection could not be performed by intention-to-treat due to the presence of a sequence effect, with a significant interaction (*p* = 0.03) between the use of VR and the period (first vs. second injection).

Through per-protocol analysis, there was no significant difference in pain scores (*p* = 0.457). Median pain score during the first injection was 3 [1; 6] vs. 4 [2; 6] in groups 1 vs. 2, respectively, and 5 [3; 7] vs. 5 [2; 7] during the second injection in groups 1 vs. 2, respectively.

### 3.3. Secondary Outcome

**Figure 3:**
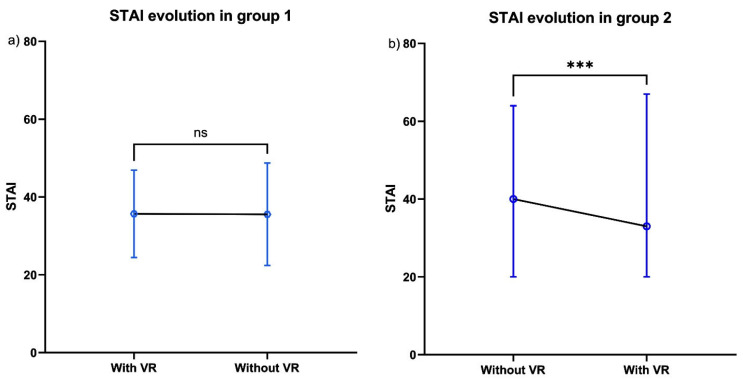
Secondary outcome. (**a**) STAI evolution in group 1. (**b**) STAI evolution in group 2. Box plot illustrating the progression of anxiety levels according to the STAI. STAI score ranging from 20 (indicating minimal anxiety) to 80 (indicating maximal anxiety). ns: not significant. *** significant difference.

Through intention-to-treat analysis, use of the VR headset during the first injection was associated with a significant reduction in STAI scores (group 1, median anxiety score 34 [28; 44] vs. 41.5 [30; 40] in group 2, and, during the second injection, median 35 [25; 27] in group 1 vs. 33.5 [27; 43] in group 2). Cross-over thus showed a significant reduction in anxiety (*p* = 0.004) (Figure 3).

Through per-protocol analysis, the median STAI score was 34 [28; 43] during the first injection, and 35 [25; 44] during the second injection for group 1. Respective scores for group 2 were 43 [30; 47] and 33 [26; 42]; cross-over showed a significant difference (*p* = 0.002).

### 3.4. Safety

While using the VR headset, 29 of the 105 patients with available data (27.6%) experienced one or more transient adverse effects. Among these, 12 patients felt visual/ocular fatigue and/or blurred vision; 11 patients had asthenia/sleepiness; 6 patients had difficulty concentrating; 3 patients had headache; 2 patients reported discomfort; 2 patients had condensation inside the headset; and 1 patient felt nauseous. None of these adverse effects interrupted the session. All adverse effects were transient and mild.

The hypnosis session offered by the VR headset was followed by 42 patients (39.25%) in group 1, and 43 patients (40.19%) in group 2.

### 3.5. Satisfaction

The overall patient satisfaction had a median of 9 (range 8–10) in group 1 and 9 (range 7–10) in group 2.

Among the 106 patients included with available data who used the headset, 92.4% would use it again for other medical procedures.

## 4. Discussion

This study shows that the use of a VR headset is associated with a significant reduction in patient anxiety during epidural steroid injection via the sacral hiatus in patients with lumbar radiculopathy. VR is a non-pharmacological approach to reducing pain and anxiety during therapeutic procedures in rheumatology and contributes to improving overall management. VR is easy and practical to use. It is less of a constraint than pharmacological treatment, and can help to limit allergies, adverse effects, and the potential for misuse that are associated with certain drugs [25]. Furthermore, any premedication has to be prescribed and taken in advance of the injection, requiring a longer duration of action. Finally, the use of MEOPA requires the presence of a specially trained staff member during the procedure. On a larger scale, conducting a subgroup analysis to assess whether some patients (age, sex, depressed, for example) respond more favorably when using a virtual reality headset would be of interest.

We observed a reduction of 2 points on the numerical rating scale, which was considered statically significant with intention-to-treat analysis, and clinically meaningful. To our knowledge, only one study somewhat similar [26] to ours used a virtual reality headset for epidural infiltration with three randomization groups (sedation group vs. virtual reality group vs. control group). The primary outcome was the verbal pain scale (0–10). They also calculated their sample size based on a reduction of at least 2 points.

There was a significant decrease in pain in both the virtual reality headset group and the sedation group compared to the control group. However, there was no significant difference between the virtual reality headset group and the sedation group.

Note that this was not a cross-over study. Another anxiety scale was also used (HADS).

The majority of infiltrations were performed transforaminally, not through the hiatus.

The findings of a recent meta-analysis that included 92 randomized trials, totaling 7133 participants, reported that there was a significant reduction in pain scores with VR across all medical procedures, albeit with significant statistical heterogeneity [27].

One possible explanation for the reduction in pain scores with the use of the VR headset may be reduced activities in the areas of the brain that are habitually active during pain, namely the insula and thalamus [28]. In addition, emotions have an influence over the mechanism of pain [29], and the VR headset acts as a distraction [30].

Regarding safety, a total of 27.6% of the 105 patients who used VR in our study suffered minor adverse effects that do not contraindicate the use of the headset. All the side effects lasted less than a minute and were mild. No medication introduction was necessary following these brief adverse effects. Indeed, this is underlined by the fact that 92.4% of the participants reported that they would use the headset again during other injections.

Moreover, the high satisfaction rate indicates that these adverse events were not perceived negatively by the patients. Reported rates of adverse effects in the literature vary between 5 and 20% [27]. The slightly higher rate observed here could be explained by a recording bias, which may overestimate frequency. In fact, the patient could check a pre-defined list of adverse effects. We should have included an open-ended question, but we were concerned that it would complicate the statistical analysis due to the multiplication of potentially different words. The types of events reported in our study were similar to those found in the literature, including nausea, headache, visual troubles, and difficult concentrating [27].

To be more precise on this subject, a more scientifically relevant questionnaire regarding satisfaction could be used.

Other possible disadvantages of VR include the cost of the headset. Further studies are warranted to confirm these encouraging, albeit preliminary, results, and to evaluate the economic impact that the use of VR headsets could have as compared to pharmacological treatment or MEOPA, and, finally, also to evaluate the feasibility of VR use by rheumatologists in private practice.

Our study has some limitations. Firstly, it was an open-label study. Secondly, only the first injection was analyzed regarding pain, while a cross-over was planned due to a sequence effect. Indeed, there was a significant sequence effect (treatment and period) in ITT for VNRS (pain). The analysis of VNRS in ITT therefore stopped at this stage. Concerning STAI (anxiety), there was no sequence effect in ITT, so we could continue with the cross-over analysis, which demonstrated a significant treatment effect. The sequence effect prevented the interpretation of certain results. In future studies, it would be interesting to conduct a larger cohort with two groups (one group using virtual reality headsets and one group without). This would allow us to completely eliminate the sequence effect that could be induced by the cross-over design.

Thirdly, adjustments were not performed due to the small sample size, and other potential biases should be considered (such as the use of anxiolytics, antidepressants, and baseline anxiety level). In future studies, it would be interesting to take these potential confounding factors into account. However, randomization made the groups comparable, thereby helping to limit these biases.

Fourthly, there is no clinically validated threshold in clinical practice for classifying anxiety using STAI scores. Various thresholds have been proposed in the literature, notably a cut-off of 39 or 50 points, but other, higher thresholds (54–55) have also been suggested [23]. Finally, the rate of missing data may have contributed to reduced statistical power, perhaps explaining the lack of statistical significance by per-protocol analysis in terms of pain scores. Conversely, the strengths of this study include its originality and methodology, notably the randomized, prospective, superiority design.

## 5. Conclusions

VR can significantly reduce pain and anxiety during epidural steroid injection via the sacral hiatus, with an acceptable tolerance and safety. Our study demonstrated a 2-point on the numerical rating scale reduction in pain level during the first injection performed under a virtual reality headset.

With respect to the pain outcome, a sequence effect was identified, which precluded the statistical analysis of the second injection.

There is a 7.5-point decrease in STAI between group 1 and group 2 during the first injection, and a 1.5-point decrease between group 1 and group 2 during the second injection. New cohort studies with larger sample sizes could help confirm these encouraging preliminary results.

## Figures and Tables

**Table 1:** Characteristics of study population.

		Group 1 n = 55	Group 2 n = 54	Available Data n = 109
Females—n (%)		31 (56.3)	28 (51.8)	(109) 100%
Age, years—median (Q1–Q3)		61 (52–69)	54.5 (44–70)	(109) 100%
Symptom duration, months—median (Q1–Q3)		6 (3–13)	6 (2–12)	(109) 100%
Pain at inclusion, by NRS—median (Q1–Q3)		6 (4–8)	6 (4–8)	(109) 100%
STAI before the injection—median (Q1–Q3)		37 (34–49)	40.5 (34–49)	(106) 97.2%
Employment status n (%)	Unemployed	9 (16.3)	8 (14.8)	(109) 100%
	Retired	24 (43.6)	18 (33.3)	
	Employed	22 (40)	28 (51.8)	
First injection—n (%)		39 (70.9)	37 (68.5)	(108) 99%
Practiced sophrology—n (%)		4 (7.2)	4 (7.4)	(107) 98%
Practiced hypnosis—n (%)		4 (7.2)	4 (7.4)	(107) 98%
Antidepressants—n (%)		8 (14.5)	10 (18.5)	(108) 99%
Anxiolytics—n (%)		7 (12.7)	7 (12.9)	(107) 98%
VR environment chosen—n (%)	Ocean	13 (23.6)	12 (22.2)	100 (91%)
	Zen garden	3 (5.4)	10 (18.5)	
	Snowy mountain	13 (23.6)	8 (14.8)	
	Sunny mountain	2 (3.6)	1 (1.8)	
	Beach	12 (21.8)	5 (9.2)	
	Forest	12 (21.8)	9 (16.6)	

## Data Availability

Data is contained within the article or Supplementary Material.

## Supplementary Materials

The following supporting information can be downloaded at https://www.mdpi.com/article/10.3390/healthcare13182376/s1. Reference [31] is cited in the supplementary materials.

## Abbreviations

The following abbreviations are used in this manuscript:

VR	Virtual reality
STAI	State Trait Anxiety Inventory
VNRS	Verbal numeric rating scale
MEOPA	Equimolar mixture of oxygen and nitrous oxide
